# Current management of gynecologic cancer in pregnancy

**DOI:** 10.4274/jtgga.2018.0044

**Published:** 2018-06-04

**Authors:** Christos Iavazzo, Evelyn Eleni Minis, Ioannis D. Gkegkes

**Affiliations:** 1 Department of Gynecological Oncology, Metaxa Cancer Hospital, Piraeus, Greece; 2 Department of Surgery, General Hospital of Attica “KAT”, Athens, Greece

**Keywords:** Gynecologic cancer, pregnancy, breast cancer, endometrial cancer, vulvar cancer, cervical cancer

## Abstract

Cancer during pregnancy is a particularly challenging complication. The incidence has increased in recent years due to childbearing at advanced maternal ages due to career choices and/or the development of reproductive technology. Approximately two thirds of cancer cases during pregnancy comprise invasive cervical cancers and breast cancer. Cancer during gestation is characterized by a need for specialized treatment due to major changes in the hormonal profile (estrogen-progesterone), metabolism (enhancement of anabolism), hemodynamic changes (hyperdynamic circulation), immunologic changes (cell-mediated and humoral immunity), and increased angiogenesis (increased blood flow towards the uterus). Moreover, the management of such patients is based on the trimester of pregnancy, type and stage of cancer, and informed consent of the mother based on her wishes. The optimal treatment of cancer during pregnancy remains elusive because there are limited data from retrospective studies with small samples. As a result, it is crucial that data regarding survival of the women and long-term follow-up of the children from different cancer centers and registries are shared. This need is dictated by the fact that the incidence of cancer during pregnancy will continue to rise as child-bearing age continues to increase.

## Introduction

The incidence of cancer during pregnancy is between 1/1000-1/1500 gestations (1). Importantly, the incidence has increased in recent years due to childbearing at advanced maternal age due to career choices and/or the development of reproductive technology. Approximately one third of cancer cases during pregnancy comprise invasive cervical cancers, another third of hematologic malignancies (lymphoma, leukemia), and the remaining third is mostly breast cancer (2). Cancer during gestation is characterized by a need for specialized treatment due to major changes in the hormonal profile (estrogen-progesterone), metabolism (enhancement of anabolism), hemodynamic changes (hyperdynamic circulation), immunologic changes (cell mediated and humoral immunity), and increased angiogenesis (increased blood flow towards the uterus) (3). Moreover, the management of such patients is based on the trimester of pregnancy, type and stage of cancer, and informed consent of the mother based on her wishes.

The aim of our narrative review was to discuss the current management of pregnant women who are diagnosed as having cancer based on the available current literature.

## Methods

### Data sources

An extensive electronic search was performed in PubMed (02/04/2018) and Scopus (02/04/2018). The adopted search strategy included the combination of the following keywords: cancer/carcinoma and/or pregnancy/gestation and management/treatment. In order to retrieve additional studies, the references of the included studies were also searched. Studies written in languages other than English were not included. The literature search had a limitation in the search range, only studies written after 1990 were considered eligible for this review. Eighteen studies were eligible to be included in our review. Studies reporting data on management of patients with cancer during pregnancy were regarded as eligible for this review. Abstracts, conference papers, book chapters, animal studies, commentaries, editorials, as well as review articles were excluded from this review.

## Breast cancer in pregnancy

Breast cancer is thought to be associated with gestation if it is diagnosed during or within one year of pregnancy. The incidence is 1/3000 gestations, and the average age at which it presents is 32-38 years (4). Importantly, it has been shown that the prognosis is worse if the diagnosis is made during lactation. The physiologic changes in the morphology of the mammary glands (engorgement of the breast) during pregnancy contribute to delayed diagnosis. Cancer during gestation can be diagnosed through mammogram; however, the false negative rate is high (around 25%) due to the increased density of the breast. For this reason, a biopsy of any palpable mass is essential to achieve early diagnosis. The level of radiation in diagnostic mammography is too low to harm the embryo. In addition, ultrasonography (USG)-guided biopsy is of paramount importance for diagnosis. Genetics play a major role, especially in BRCA1 and BRCA2 carriers.

Regarding treatment, the literature does not suggest a better prognosis if a pregnancy is terminated (5,6). Treatment is the same as in patients who are not pregnant. Small tumors are treated by lumpectomy, whereas larger tumors are managed by modified radical mastectomy and axillary lymph node dissection. In recent years, the role of the sentinel lymph node has also been under consideration in order to minimize the extent of dissection. According to Balaya et al. (7), the blue dye injection has a theoretical 2% anaphylactic shock rate. However, Tc-99m injection at a dose of 12.1-18.5 mBq is safe for the fetus and the obstetric outcome. Adjuvant chemotherapy can be initiated after 16 weeks’ gestation, following the completion of organogenesis, while radiation is delayed until after delivery, despite evidence that radiation of the axilla or chest could be safe after the 1^st^ trimester (8,9). External beam radiation can be used whenever the fetus can be exposed to secondary radiation due to head leakage, scatter from the machine, and scatter produced inside the patient (10). Appropriate selection of irradiation parameters and different shielding devises can minimize the risk. It seems that overall survival of patients with breast cancer diagnosed during pregnancy is worse compared with prior pregnancy controls as a principal result of a possible delayed diagnosis. The 5-year survival depends on cancer stage ranging from 85% in stage I down to 5% in stage IV (9).

## Vulvar and vaginal pre-invasive and invasive lesions

Warts and intraepithelial lesions of the squamous epithelium (SIL) tend to increase in size during pregnancy. In most cases, no treatment is recommended unless the lesions are symptomatic (11). These lesions regress following delivery. Alternative forms of treatment are imiquimod (Aldara^TM^), 5-FU, trichloroacetic acid, podophyllin, removal by laser ablation, surgical removal, loop electrosurgical excision procedure (LEEP) or cryotherapy (12).

Diagnosis of vulvar cancer during pregnancy is especially rare because it is more common in postmenopausal women, with the average age of diagnosis being 60-70 years. However, the literature describes 37 cases of vulvar cancer diagnosed during pregnancy (13,14). A systematic review of the literature shows that the mean age during diagnosis was 30.7 years old, three out of four women were diagnosed as having vulvar mass/swelling and more than 50% during the second trimester. Squamous histology was found in the majority of cases (13). Surgical removal is recommended (wide excision with unilateral or bilateral inguinal lymph node dissection) during the second or third trimester, more commonly before 36 weeks’ gestation, in order for the wound to heal prior to delivery. Importantly, hemostasis is often challenging due to increased vascularity. The majority of cases result in excellent pregnancy outcomes; however, delayed diagnosis and management affect disease-free and overall survival (13).

The management of vulvar cancer in pregnancy does not differ from that in a non-pregnant patient and consists of radical vulvectomy and groin lymph node dissection when the depth of invasion is >1 mm. A systematic review concluded that in 72% of cases, the preferred procedure by gynecologic oncologists was radical vulvectomy, which was usually performed post-partum (59.3%). Less commonly, the procedure may also be performed during the second trimester, although only one patient underwent surgery in the first trimester (15). The majority of patients (95%) underwent bilateral inguinal femoral node dissection in the postpartum period. Regarding sentinel node biopsy with Tc-99m (no blue dye), only one such case has been reported in which there was no harm to the fetus (16).

## Pre-invasive and invasive lesions of the cervix during pregnancy

The prevalence of human papilloma virus in women aged from 14 to 59 years is 42.5% (17). Regarding pre-invasive lesions of the cervix, 2-6.5% of cervical intraepithelial neoplasia (CIN)/squamous intraepithelial lesion (SIL) cases present during gestation; 10-50 cases of cancer have been reported per 100,000 gestations (18). In addition, 1.9% of microinvasive cancer cases are diagnosed during pregnancy (19). Pap smears as well as colposcopy should be performed as a routine examination for the diagnosis of pre-invasive lesions during pregnancy. During pregnancy, hypertrophy of the glandular epithelium is observed, resulting in the translocation of the transformation zone to the ectocervix. In addition, the increased vascularity, engorged cervix, and glandular hyperplasia make diagnosis through cytology and colposcopy challenging. There is a characteristic purplish hue due to the increased vascularity as well as a hypertrophy of the glandular epithelium and edema of the cervix. However, indications for colposcopy remain the same in pregnancy.

According to the literature, 0.4-10% of CIN II-III cases develop into invasive cancer (20). Conversely, 47-74% of cases regress (20,21,22). A conservative approach of pre-invasive lesions is necessary in order to avoid complications such as preterm labor. As a result, the patient should be monitored every 6-8 weeks, colposcopy should be performed every 12 weeks, and the patient should be reassessed 12 weeks after delivery (23,24). Treatment should be delayed until after delivery. During pregnancy, cone biopsy is recommended in patients who are suspected to have invasive cancer. However, an Israeli study suggested that LEEP during the first 15 weeks of gestation was safe and reconsideration of guidelines was proposed (25). More specifically, with a non-satisfactory colposcopy/Pap smear, adenocarcinoma in situ and microinvasive cancer are indications for a cone biopsy (24). Cone biopsy is performed during the 14-20^th^ weeks of gestation. Postoperative complications include miscarriage (5%) and bleeding (9%) (24). Diagnostic examinations include magnetic resonance imaging (MRI) of the abdomen (use of computed tomography is limited during pregnancy), chest X-ray, carcinoembryonic antigen levels, cystoscopy, and rectal colonoscopy in order to achieve a complete staging. Counseling by a gynecologist oncologist is also recommended.

The appropriate treatment is often individualized and depends on whether the patient wishes to conserve her fertility, staging, and gestational age. More specifically, continuation of pregnancy until fetal lung maturity is achieved is suggested in stage I disease at 20 weeks’ gestation and over. For stage IA1, cone biopsy is preferred with no additional treatment required (1.2% risk of lymph node metastases), with vaginal delivery being the preferred mode of delivery (23). For stage IA2, cone biopsy combined with pelvic lymph node dissection during pregnancy and excision of the cervix immediately following delivery is recommended (19). With regards to stage IA2 and IIA, a C-section followed by radical hysterectomy and lymph node dissection is recommended. Often, transposition of the ovaries out of the range of radiation, in order to conserve ovarian function is considered (26).

In a recent study by Vercellino et al. (27), laparoscopic pelvic lymphadenectomy was performed on 32 patients in their first and second trimester. The median number of excised lymph nodes was 14 (range, 8-57), and it was concluded that the optimal time for operation was prior to 22-24 weeks’ gestation. In addition, no intraoperative complications were reported.

Radiation is the first choice in stage IIB or above (26). Prognosis of cervical cancer is not affected by gestation. Twenty percent of cases are diagnosed due to post-coital bleeding, and 63% do not present with an abnormal Pap smear (19). Mortality is low, and the survival rate is up to 95%.

In a recent cohort study by Bigelow et al. (28), a planned C-section was the preferred delivery method in cases of confirmed malignancy; vaginal deliveries performed on patients with microinvasive cancer or an unconfirmed diagnosis were not shown to affect disease progression and survival or cause perinatal complications. Despite these results, the authors concluded that elective C-section could be suggested as the optimal management, in view of potential local recurrence and distal metastasis (29,30,31,32,33).

The same study reported that survival was not affected in cases were surgical intervention was performed in the post-partum period, reporting 5-year survival rates comparable to those of large-population studies (34). Pregnant patients were not found to have any difference regarding oncologic outcome, despite the considerable delay between diagnosis and intervention. Thus, the authors suggested delaying delivery to a gestational age of 37-39 weeks, especially considering the significant morbidity and mortality associated with preterm delivery (35,36,37,38). Conversely, a number of recent publications regarding cervical cancer in pregnancy suggested preterm delivery in order to initialize treatment earlier (19,35,39). In addition, in a study by Xia et al. (33), delayed intervention was associated with decreased survival. However, this study group included a significant number of patients with malignant disease >4 cm and aggressive histopathology (33). Similar findings have not been reported by other studies (40,41,42).

Regarding chemotherapy, a platinum-based cisplatin is the preferred agent (50-100 mg/m^2^) (19). This may be as a monotherapy or combined with paclitaxel (175 mg/m^2^), bleomycin, vincristine, 5-fluorouracil or vincristine and bleomycin. Another combination that has been suggested is paclitaxel and carboplatin (43,44). It has been reported that chemotherapy should be administered every three weeks, and delivery should be scheduled at a minimum of three weeks following the final dose, in order to minimize risks of perinatal complications (45,46). Regarding intrauterine complications, it is suggested that chemotherapy should not be administered during the first trimester, due to risks of miscarriage and fetal malformation (47). More specifically, in the case of monotherapy, there is a 7.5-17% risk, and combination therapy is associated with a 25% risk (48).

The risks of chemotherapy are immediately correlated with gestational age. Exposure in weeks 1-2 (implantation phase) causes lethal mutations due to a direct effect on stem cells, and exposure between weeks 2 and 8 (organogenesis) affects the heart, limbs, and neural tube. Finally, exposure after week 8 endangers central nervous system (CNS) development as well (49). Conversely, a recent study by Köhler et al. (50) on 21 pregnant women undergoing chemotherapy with cisplatin reported no malformations or incidents of perinatal morbidity (50). Another study by Köhler et al. (50) found that levels of platinum in umbilical cord blood and amniotic fluid were lower than those in maternal blood. The authors formulated the hypothesis that the placenta provides a possible filtration mechanism.

Data on neonatal outcomes, following chemotherapy during the later stages of gestation are scarce, especially regarding long-term follow-up (51). One study by Amant et al. (52) on 70 patients followed up for a median of 22.3 months reported no adverse incidents regarding cognitive function. None of these cases included treatment in the first trimester after being exposed in utero to chemotherapy (52). Only 25 cases of neoadjuvant chemotherapy during stage IB1 have been documented. Again, there are few studies reporting long-term follow-up (51,53,54,55,56).

## Endometrial cancer during pregnancy

The incidence of endometrial cancer in women aged below 40 years is very low. The majority of such patients are obese and diagnosed as having grade 1, stage I disease; however, women with low body mass index (BMI) (<25 kg/m^2^) are more likely to have more aggressive tumors (clear cell or serous papillary) and/or more advanced stage (57). Gestation is a state of naturally increased progesterone, which acts protectively on the endometrium. It has been hypothesized that endometrial cancer during pregnancy might be due to an immature, progesterone-resistant endometrium. Malignancy may originate from immature basal cells, irresponsive to hormonal stimulation (58). The literature describes 31 cases of endometrial cancer stage I during pregnancy (59). Furthermore, fertility-sparing progestin therapy (oral medroxyprogesterone or/and levonorgestrel intrauterine system) is quite common nowadays in young nulliparous women. Park et al. (60) showed that pre- and post-treatment BMI <25 kg/m^2^ could positively affect treatment response and recurrence rates.

## Adnexal masses during pregnancy

The total incidence of adnexal masses during pregnancy is 1:500 gestations, and the incidence of ovarian cancer is 1:10,000-1:50,000 gestations (61,62). Adnexal masses are usually found incidentally during C-section (1/200-400 C-sections) (63). Of these, 33% are non-neoplastic (luteal cysts), 63% are benign (dermoid cyst 36%, serous cystadenoma 17%, mucinous cystadenoma 8%), 3% are malignant-low malignant potential and adenocarcinoma, and stromal or sex cord tumors comprise 1% (64). Germ cell tumors are more frequent in younger patients (65). However, the incidence of epithelial ovarian cancer is increasing as maternal age is also on the rise. The diagnostic tests for ovarian cancer during gestation include pelvic USG, MRI of the abdomen, chest X-ray, CA-125 (despite the fact that levels increase during pregnancy and normalize after 12 weeks gestation), alpha-fetoprotein (AFP), beta-human chorionic gonadotropin, lactase dehydrogenase, liver function tests, urea, creatinine, and intraoperative biopsy also plays a significant role.

Tumors of low malignant potential and non-epithelial tumors (e.g. sex cord tumors) are usually diagnosed at an early stage (stage I) (66), for which bilateral salpingo-oophorectomy, omentectomy and cytology at 16-18 weeks are recommended. Epithelial ovarian cancer at stage IA is treated with unilateral salpingo-oophorectomy, omentectomy, and cytology at 16-18 weeks. Further treatment is not needed and gestation can proceed safely. With regards to epithelial ovarian cancer stages IC-IV, chemotherapy should be delayed until after 12-16 weeks’ gestation, and excision of the corpus luteum should be delayed until after 14 weeks’ gestation (45,67). If diagnosis is made during the first trimester, pregnancy termination is recommended, followed by treatment. If the diagnosis is made during the second or third trimester, chemotherapy is administered (platine-paclitaxel) initially (63). After fetal lung maturity is achieved, C-section is performed followed by surgical tumor debulking.

In the event of suspected or confirmed cancer, surgical staging is recommended. Epithelial ovarian cancer standard treatment includes total hysterectomy, bilateral salpingo-oophorectomy, optimal debulking, followed by 6 cycles of combined carboplatin and paclitaxel (68). However, ovarian cancer during pregnancy may be treated more conservatively with ovarian cystectomy or unilateral salpingo-oophorectomy, including biopsies. Occasionally treatment will also include omentectomy, appendectomy, peritoneal biopsies, and pelvic and para-aortic lymphadenectomy (69). The aforementioned conservation of the contralateral ovary and uterus may be considered in stage IA, grade 1 to 2, following surgical staging, when histology is non-clear cell (70). Following the above, if the patient desires to continue the pregnancy, chemotherapy may be either delayed until after fetal lung maturity is achieved, and initiated after delivery, or administered neoadjuvantly (68,70). For stage III or IV, treatment varies by trimester of gestation. In the first trimester, if conservation of pregnancy prevents optimal debulking, the pregnancy must be terminated due to the risks of chemotherapy treatment. During the second trimester, the optimal treatment is unilateral or bilateral oophorectomy, surgical excision of peritoneal tumors, omentectomy, and pelvic and para-aortic lymph node sampling and appendectomy. The above must be followed by initiation of chemotherapy and term C-section and hysterectomy. Finally, in the third trimester, chemotherapy after C-section, hysterectomy, and surgical staging are indicated (70). Several studies have reported on delaying completion of debulking until a few weeks following vaginal delivery and administering cycles of platinum-based chemotherapy. Conversely, other authors recommend a C-section after fetal lung maturity is achieved (69).

Regarding neonatal outcomes, a number of studies have shown favorable outcomes following treatment with carboplatin and paclitaxel combined during pregnancy. As mentioned above, chemotherapy is not administered during the first trimester due to the risk associated with the treatment. More specifically, teratogenesis may occur in up to 25% of cases of carboplatin treatment; the risk may be as low as 1.3% if treatment is in the second and third trimesters (71). The prognosis of ovarian cancer is not affected by gestation.

Cancer during pregnancy is a particularly challenging complication and the optimal treatment remains elusive because there are limited data from retrospective studies with small samples. As a result, it is crucial that data regarding survival of the women and long-term follow up of the children from different cancer centers and registries are shared. This need is dictated by the fact that the incidence of cancer during pregnancy will continue to rise as child-bearing age continues to increase.

## References

[1] Esposito S, Tenconi R, Preti V, Groppali E, Principi N (2016). Chemotherapy against cancer during pregnancy: A systematic review on neonatal outcomes. Medicine (Baltimore).

[2] Mahmoud HK, Samra MA, Fathy GM (2016). Hematologic malignancies during pregnancy: A review. J Adv Res.

[3] Weisz B, Meirow D, Schiff E, Lishner M (2004). Impact and treatment of cancer during pregnancy. Expert Rev Anticancer Ther.

[4] PDQ Adult Treatment Editorial Board (2002-2017 Dec 22.). Breast Cancer Treatment During Pregnancy (PDQ®): Health Professional Version. PDQ Cancer Information Summaries [Internet]. Bethesda (MD). National Cancer Institute (US).

[5] Basta P, Bak A, Roszkowski K (2015). Cancer treatment in pregnant women. Contemp Oncol (Pozn).

[6] Keleher AJ, Theriault RL, Gwyn KM, Hunt KK, Stelling CB, Singletary SE, et al (2002). Multidisciplinary management of breast cancer concurrent with pregnancy. J Am Coll Surg.

[7] Balaya V, Bonsang-Kitzis H, Ngo C, Delomenie M, Gosset M, Mimouni M, et al (2018). What about sentinel lymph node biopsy for early breast cancer during pregnancy?. J Gynecol Obstet Hum Reprod.

[8] Sasidharan R, Harvey V (2010). Pregnancy and breast cancer. Obstet Med.

[9] Biglia N, Alessandro Peccatori F (2015). Breast Cancer, Fertility Preservation and Reproduction. Springer International Publishing.

[10] Mazonakis M, Damilakis J (2017). Estimation and reduction of the radiation dose to the fetus from external-beam radiotherapy. Phys Med.

[11] Tseng JY, Bastu E, Gungor-Ugurlucan F (2012). Management of precancerous lesions prior to conception and during pregnancy: a narrative review of the literature. Eur J Cancer Care (Engl).

[12] Yang LJ, Zhu DN, Dang YL, Zhao X (2016). Treatment of condyloma acuminata in pregnant women with cryotherapy combined with proanthocyanidins: Outcome and safety. Exp Ther Med.

[13] Matsuo K, Whitman SA, Blake EA, Conturie CL, Ciccone MA, Jung CE, et al (2014). Feto-maternal outcome of pregnancy complicated by vulvar cancer: a systematic review of literature. Eur J Obstet Gynecol Reprod Biol.

[14] Hasanzadeh M, Zamiri-Akhlaghi A, Hassanpoor-Moghaddam M, Shahidsales S (2014). Vulvar carcinoma in pregnant women aged less than 40 years: case report. Iran J Cancer Prev.

[15] Matsuo K, Whitman SA, Blake EA, Conturie CL, Ciccone MA, Jung CE et al (2014). Feto-maternal outcome of pregnancy complicated by vulvar cancer: a systematic review of literature. Eur J Obstet Gynecol Reprod Biol.

[16] Nijman TA, Schutter EM, Amant F (2012). Sentinel node procedure in vulvar carcinoma during pregnancy: A case report. Gynecol Oncol Case Rep.

[17] Hariri S, Unger ER, Sternberg M, Dunne EF, Swan D, Patel S, et al (2011). Prevalence of genital human papillomavirus among females in the United States, the National Health and Nutrition Examination Survey, 2003-2006. J Infect Dis.

[18] Nguyen C, Montz FJ, Bristow RE (2000). Management of stage I cervical cancer in pregnancy. Obstet Gynecol Surv.

[19] La Russa M, Jeyarajah AR (2016). Invasive cervical cancer in pregnancy. Best Pract Res Clin Obstet Gynaecol.

[20] Mailath-Pokorny M, Schwameis R, Grimm C, Reinthaller A, Polterauer S (2016). Natural history of cervical intraepithelial neoplasia in pregnancy: postpartum histo-pathologic outcome and review of the literature. BMC Pregnancy Childbirth.

[21] Paraskevaidis E, Koliopoulos G, Kalantaridou S, Pappa L, Navrozoglou I, Zikopoulos K, et al (2002). Management and evolution of cervical intraepithelial neoplasia during pregnancy and postpartum. Eur J Obstet Gynecol Reprod Biol.

[22] Yost NP, Santoso JT, McIntire DD, Iliya FA (1999). Postpartum regression rates of antepartum cervical intraepithelial neoplasia II and III lesions. Obstet Gynecol.

[23] Han SN, Mhallem Gziri M, Van Calsteren K, Amant F (2013). Cervical cancer in pregnant women: treat, wait or interrupt? Assessment of current clinical guidelines, innovations and controversies. Ther Adv Med Oncol.

[24] Gonçalves CV, Duarte G, Costa JS, Marcolin AC, Bianchi MS, Dias D, et al (2009). Diagnosis and treatment of cervical cancer during pregnancy. Sao Paulo Med J.

[25] Siegler E, Lavie O, Amit A, Vaknin Z, Auslander R, Blumenfeld Z, et al (2017). Should the Risk of Invasive Cancer in Pregnancy and the Safety of Loop Electrosurgical Excision Procedure During the First 15 Weeks Change Our Practice?. J Low Genit Tract Dis.

[26] Eitan R, Abu-Rustum NR (2003). Management of cervical carcinoma diagnosed during pregnancy. Primary care update for Ob/Gyns.

[27] Vercellino GF, Koehler C, Erdemoglu E, Mangler M, Lanowska M, Malak AH, et al (2014). Laparoscopic pelvic lymphadenectomy in 32 pregnant patients with cervical cancer: rationale, description of the technique, and outcome. Int J Gynecol Cancer.

[28] Bigelow CA, Horowitz NS, Goodman A, Growdon WB, Del Carmen M, Kaimal AJ (2017). Management and outcome of cervical cancer diagnosed in pregnancy. Am J Obstet Gynecol.

[29] Sood AK, Sorosky JI, Mayr N, Anderson B, Buller RE, Niebyl J (2000). Cervical cancer diagnosed shortly after pregnancy: prognostic variables and delivery routes. Obstet Gynecol.

[30] Cliby WA, Dodson MK, Podratz KC (1994). Cervical cancer complicated by pregnancy: episiotomy site recurrences following vaginal delivery. Obstet Gynecol.

[31] Goldman NA, Goldberg GL (2003). Late recurrence of squamous cell cervical cancer in an episiotomy site after vaginal delivery. Obstet Gynecol.

[32] Baloglu A, Uysal D, Aslan N, Yigit S (2007). Advanced stage of cervical carcinoma undiagnosed during antenatal period in term pregnancy and concomitant metastasis on episiotomy scar during delivery: a case report and review of the literature. Int J Gynecol Cancer.

[33] Xia T, Gao Y, Wu B, Yang Y (2015). Clinical analysis of twenty cases of cervical cancer associated with pregnancy. J Cancer Res Clin Oncol.

[34] SEER. Stat fact sheet: cervix uteri cancer. Available at:.

[35] Greer BE, Easterling TR, McLennan DA, Benedetti TJ, Cain JM, Figge DC, et al (1989). Fetal and maternal considerations in the management of stage I-B cervical cancer during pregnancy. Gynecol Oncol.

[36] Engle WA, Tomashek KM, Wallman C, Committee on Fetus and Newborn, American Academy of Pediatrics (2007). "Late-preterm" infants: a population at risk. Pediatrics.

[37] Leone A, Ersfeld P, Adams M, Schiffer PM, Bucher HU, Arlettaz R (2012). Neonatal morbidity in singleton late preterm infants compared with full-term infants. Acta Paediatr.

[38] Sengupta S, Carrion V, Shelton J, Wynn RJ, Ryan RM, Singhal K, et al (2013). Adverse neonatal outcomes associated with early-term birth. JAMA Pediatr.

[39] Mogos MF, Salemi JL, Sultan DH, Shelton MM, Salihu HM (2015). Trends in Cervical Cancer Among Delivery-Related Discharges and its Impact on Maternal-Infant Birth Outcomes (United States, 1998-2009). Open Nurs J.

[40] Zemlickis D, Lishner M, Degendorfer P, Panzarella T, Sutcliffe SB, Koren G (1991). Maternal and fetal outcome after invasive cervical cancer in pregnancy. J Clin Oncol.

[41] Germann N, Haie-Meder C, Morice P, Lhomme C, Duvillard P, Hacene K, et al (2005). Management and clinical outcomes of pregnant patients with invasive cervical cancer. Ann Oncol.

[42] Kärrberg C, Rådberg T, Holmberg E, Norström A (2015). Support for down-staging of pregnancy-associated cervical cancer. Acta Obstet Gynecol Scand.

[43] Zagouri F, Sergentanis TN, Chrysikos D, Bartsch R (2013). Platinum derivatives during pregnancy in cervical cancer: a systematic review and meta-analysis. Obstet Gynecol.

[44] Robova H, Halaska MJ, Pluta M, Skapa P, Matecha J, Lisy J, et al (2014). Oncological and pregnancy outcomes after high-dose density neoadjuvant chemotherapy and fertility-sparing surgery in cervical cancer. Gynecol Oncol.

[45] Amant F, Halaska MJ, Fumagalli M, Dahl Steffensen K, Lok C, Van Calsteren K, et al (2014). Gynecologic cancers in pregnancy: guidelines of a second international consensus meeting. Int J Gynecol Cancer.

[46] Brewer M, Kueck A, Runowicz CD (2011). Chemotherapy in pregnancy. Clin Obstet Gynecol.

[47] Cardonick E, Iacobucci A (2004). Use of chemotherapy during human pregnancy. Lancet Oncol.

[48] Weisz B, Schiff E, Lishner M (2001). Cancer in pregnancy: maternal and fetal implications. Hum Reprod Update.

[49] Schoenwolf GC, Bleyl SB, Brauer PhR, et al (2008). Larsen's human embryology. 4th ed.

[50] Köhler C, Oppelt P, Favero G, Morgenstern B, Runnebaum I, Tsunoda A, et al (2015). How much platinum passes the placental barrier? Analysis of platinum applications in 21 patients with cervical cancer during pregnancy. Am J Obstet Gynecol.

[51] Amant F, Vandenbroucke T, Verheecke M, Fumagalli M, Halaska MJ, Boere I, et al (2015). Pediatric Outcome after Maternal Cancer Diagnosed during Pregnancy. N Engl J Med.

[52] Amant F, Van Calsteren K, Halaska MJ, Gziri MM, Hui W, Lagae L, et al (2012). Long-term cognitive and cardiac outcomes after prenatal exposure to chemotherapy in children aged 18 months or older: an observational study. Lancet Oncol.

[53] Marnitz S, Köhler C, Oppelt P, Schmittel A, Favero G, Hasenbein K, et al (2010). Cisplatin application in pregnancy: first in vivo analysis of 7 patients. Oncology.

[54] Fruscio R, Villa A, Chiari S, Vergani P, Ceppi L, Dell'Orto F, et al (2012). Delivery delay with neoadjuvant chemotherapy for cervical cancer patients during pregnancy: a series of nine cases and literature review. Gynecol Oncol.

[55] Ayhan A, Dursun P, Karakaya BK, Ozen O, Tarhan C (2012). Neoadjuvant chemotherapy followed by cesarean radical hysterectomy in a triplet pregnancy complicated by clear cell carcinoma of the cervix: a case presentation and literature review. Int J Gynecol Cancer.

[56] Gambino A, Gorio A, Carrara L, Agoni L, Franzini R, Lupi GP, et al (2011). Cancer in pregnancy: maternal and fetal implications on decision-making. Eur J Gynaecol Oncol.

[57] Duska LR, Garrett A, Rueda BR, Haas J, Chang Y, Fuller AF (2001). Endometrial cancer in women 40 years old or younger. Gynecol Oncol.

[58] Hoellen F, Reibke R, Hornemann K, Thill M, Luedders DW, Kelling K, et al (2012). Cancer in pregnancy. Part I: basic diagnostic and therapeutic principles and treatment of gynecological malignancies. Arch Gynecol Obstet.

[59] Ilancheran A, Low J, Ng JS (2012). Gynaecological cancer in pregnancy. Best Pract Res Clin Obstet Gynaecol.

[60] Park JY, Seong SJ, Kim TJ, Kim JW, Bae DS, Nam JH (2017). Significance of body weight change during fertility-sparing progestin therapy in young women with early endometrial cancer. Gynecol Oncol.

[61] Cavaco-Gomes J, Jorge Moreira C, Rocha A, Mota R, Paiva V, Costa A (2016). Investigation and Management of Adnexal Masses in Pregnancy. Scientifica (Cairo).

[62] Marret H, Lhommé C, Lecuru F, Canis M, Lévèque J, Golfier F, et al (2010). Guidelines for the management of ovarian cancer during pregnancy. Eur J Obstet Gynecol Reprod Biol.

[63] Schwartz N, Timor-Tritsch IE, Wang E (2009). Adnexal masses in pregnancy. Clin Obstet Gynecol.

[64] Owens GL, Kitchener HC (2016). Premalignant disease in the genital tract in pregnancy. Best Pract Res Clin Obstet Gynaecol.

[65] Iavazzo C, Vorgias G, Iavazzo PE, Gkegkes ID (2017). Fertility sparing approach as the standard of care in young patients with immature teratomas. J Turk Ger Gynecol Assoc.

[66] Iavazzo C, Gkegkes ID, Vrachnis N (2016). Primary peritoneal cancer in BRCA carriers after prophylactic bilateral salpingo-oophorectomy. J Turk Ger Gynecol Assoc.

[67] Grimm D, Woelber L, Trillsch F, Keller-v Amsberg G, Mahner S (2014). Clinical management of epithelial ovarian cancer during pregnancy. Eur J Cancer.

[68] Stuart GC, Kitchener H, Bacon M, duBois A, Friedlander M, Ledermann J, et al (2011). 2010 Gynecologic Cancer InterGroup (GCIG) consensus statement on clinical trials in ovarian cancer: report from the Fourth Ovarian Cancer Consensus Conference. Int J Gynecol Cancer.

[69] Modares Gilani M, Karimi Zarchi M, Behtash N, Ghaemmaghami F, Mousavi AS, Behnamfar F (2007). Preservation of pregnancy in a patient with advanced ovarian cancer at 20 weeks of gestation: case report and literature review. Int J Gynecol Cancer.

[70] Minig L, Otaño L, Diaz-Padilla I, Alvarez Gallego R, Patrono MG, Valero de Bernabé J (2013). Therapeutic management of epithelial ovarian cancer during pregnancy. Clin Transl Oncol.

[71] Doll DC, Ringenberg QS, Yarbro JW (1988). Management of cancer during pregnancy. Arch Intern Med.

